# Decision-Making Tool for Planning Camera-Assisted and Awake Intubation in Head and Neck Surgery

**DOI:** 10.1001/jamaoto.2025.0538

**Published:** 2025-05-01

**Authors:** Zohal Popal, Hans-Heinrich Sieg, Lynn Müller-Wiegand, Philipp Breitfeld, Andre Dankert, Phillip B. Sasu, Viktor A. Wünsch, Linda Krause, Christian Zöllner, Martin Petzoldt

**Affiliations:** 1 Department of Anesthesiology, Center of Anesthesiology and Intensive Care Medicine, University Medical Center Hamburg-Eppendorf, Hamburg, Germany; 2 Institute for Applied Medical Informatics, University Medical Centre Hamburg-Eppendorf, Hamburg, Germany; 3 Institute of Medical Biometry and Epidemiology, University Medical Center Hamburg-Eppendorf, Hamburg, Germany

## Abstract

**Importance:**

Indication criteria for camera-assisted and awake tracheal intubation are vague. It is unknown if diagnostic and clinical data from multiple sources, such as transnasal videoendoscopy or symptoms for pharyngolaryngeal lesions, might improve preanesthesia airway management planning and decision-making in patients undergoing head and neck surgery.

**Objective:**

To develop and validate a new decision-making tool (Evidence-Based Algorithm for the Expected Difficult Intubation [Expect-It]) and show noninferiority to the clinical standard (nonalgorithm-based decision-making).

**Design, Setting, and Participants:**

This single-center study prospectively developed and validated a decision-making tool with a 2-stage design that included anesthetic cases from patients undergoing head and neck surgery between May 1, 2021, and January 29, 2022. Data were analyzed between August 2021 (first stage) and December 2023.

**Exposures:**

Airway-related risk factors from 4 domains (previous intubation difficulties, physical examination, physician’s rating of difficult airway indicators, and pharyngolaryngeal lesions/transnasal videoendoscopy findings) were preoperatively assessed. During airway management planning, physicians proposed a first-line tracheal intubation technique (camera-assisted or direct laryngoscopy) and strategy (awake or asleep tracheal intubation). In the development cohort, these proposals were nonalgorithm-based (clinical standard); in the validation cohort, they relied on the Expect-It decision-making tool.

**Main Outcomes and Measures:**

Regularized regression was used to select potentially predictive airway-related risk factors (covariables). The final decision-making tool is a combined score originating from 2 multivariable logistic regression models that predict 2 different primary outcomes: the most appropriate (1) tracheal intubation technique (camera-assisted or direct laryngoscopy) and (2) strategy (awake or asleep), as determined by the anesthesiologists after tracheal intubation.

**Results:**

Of 1201 patients (mean [SD] age, 50.3 [19.0] years; 695 [58%] male), 1282 anesthetic cases were included in the analysis: 602 in the development and 680 in the validation cohort. The area under the curve of the decision-making tool was 0.86 (95% CI, 0.81-0.90) to predict appropriate camera-assisted and 0.97 (95% CI, 0.96-0.99) to predict appropriate awake tracheal intubation in the development cohort. The sensitivity of the Expect-It tool to predict both appropriate camera-assisted and awake tracheal intubation was superior compared to the clinical standard (camera-assisted: 88% [95% CI, 81%-93%] vs 35% [95% CI, 27%-44%], respectively; awake tracheal intubation: 97% [95% CI, 81%-100%] vs 29% [95% CI, 15%-50%], respectively), and specificity was noninferior to the clinical standard (camera-assisted: 97% [95% CI, 96%-98%] vs 96% [95% CI, 93%-97%], respectively; awake tracheal intubation: 100% [95% CI, 99%-100%] vs 98% [95% CI, 97%-99%], respectively). After tool implementation, the first-attempt success rate increased from the development to validation cohort (437 [73%] vs 557 [82%], respectively; odds ratio, 1.72 [95% CI, 1.32-2.22]), while failed direct laryngoscopy decreased from the development to validation cohort (45 [8%] vs 10 [2%], respectively; odds ratio, 0.18 [95% CI, 0.09-0.37]).

**Conclusions and Relevance:**

In this study, the Expect-It tool for airway management planning was prospectively developed and validated. The tool was found to support airway management planning accurately and may serve as a precursor for intelligent algorithms.

## Key Points

**Question:**

Can decision-making tools that use clinical and diagnostic data from multiple sources, such as previous intubation difficulties, physical examinations, symptoms for pharyngolaryngeal lesions, and transnasal videoendoscopy, be useful for preanesthesia planning of camera-assisted and awake tracheal intubation?

**Findings:**

In this study of 1282 anesthetic cases from 1201 patients undergoing head and neck surgery, a new decision-making tool (Evidence-Based Algorithm for the Expected Difficult Intubation [Expect-It]) was prospectively developed and validated to accurately support airway management planning.

**Meaning:**

Preoperative clinical and diagnostic data from multiple sources can be systematically used by physicians or intelligent algorithms to accurately support airway management planning and might hereby avoid both indiscriminately overusing and underusing health care resources.

## Teaser

This single-center study prospectively develops and validates a new decision-making tool for airway management planning in patients undergoing head and neck surgery.

## Introduction

Camera-assisted tracheal intubation techniques such as videolaryngoscopy or flexible bronchoscopy are recommended techniques to manage difficult tracheal intubation in anesthesia, intensive care, and emergency medicine.^1,2,3,4,5,6,7,8^ Awake tracheal intubation has a favorable safety profile but might be underused.^1^ Still, indication criteria for awake tracheal intubation are vague.^1,2,4,5,9^

Existing airway risk prediction tests have a low sensitivity, which in turn might be accompanied with false-negative test results (unexpected difficult intubations).^10,11,12^ A large retrospective study found that more than 90% of difficult tracheal intubations were unexpected.^4,13^ Current airway risk prediction tests predominantly rely on anatomic assessments and jaw joint function, while pharyngolaryngeal lesions are not considered.^10,11^ Preoperative transnasal videoendoscopy might close this gap.^14,15,16^ Previous difficult airway management is the most accurate predictor of future difficulty.^6,17,18^ Recently, a prospectively developed universal classification for videolaryngoscopy, the Videolaryngoscopic Intubation and Difficult Airway Classification (VIDIAC) score, has been introduced that closes this gap.^6,19^

Traditional medical decision-making is unstructured and incorporates evidence haphazardly.^20^ Unlike risk assessment tests, decision-making tools are linked to specific therapeutic targets or preventive concepts^10,11,14^ to allow rational choices and accurate risk perceptions.^9,20,21^ Recent airway management guidelines highlighted decision-making tools for awake tracheal intubation.^2,9,22^

A decision-making tool for airway planning could either be beneficial by reducing unexpected difficult intubations (here, high sensitivity is pivotal) or by avoiding overtreatment (eg, awake tracheal intubation in nonindicated patients; here, high specificity is required). It is not certain, however, whether decision-making tools might be beneficial for airway management planning to support and complement nonalgorithm-based decision-making.

This study aimed to develop the Evidence-Based Algorithm for the Expected Difficult Intubation (Expect-It) decision-making tool for airway management planning that incorporates recordings from previous intubation difficulties, physical examination, physician’s rating of difficult airway indicators, suspected or verified pharyngolaryngeal lesions, as well as transnasal videoendoscopy findings to predict the appropriate tracheal intubation technique (camera-assisted or direct laryngoscopy) and strategy (awake or asleep) in an individual patient, and to show noninferiority to nonalgorithm-based decision-making (clinical standard). A secondary aim was to investigate the implications of the clinical implementation of the decision-making tool on clinician- and patient-centered outcomes.

## Methods

### Study Design and Participants

This single-center prospective study for the development and validation of a multivariable prediction model was conducted in accordance with the Declaration of Helsinki. Study design and reporting were carried out in accordance with the Transparent Reporting of a Multivariable Prediction Model for Individual Prognosis or Diagnosis (TRIPOD)^23^ and Standards for Reporting of Diagnostic Accuracy (STARD)^24^ reporting guidelines. The study was approved by the Ethics Committee of the Medical Association of Hamburg, and all participants provided written informed consent.

This study was designed to develop and validate a decision-making tool that uses patient-specific information from multiple sources to predict the most appropriate tracheal intubation technique and strategy preoperatively. In the future, this additional information provided by the decision-making tool could be used by physicians or intelligent algorithms to support decision-making and to adopt the preoperative strategy with foresight.

Consecutive adult patients (18 years and older) scheduled for ear, nose, and throat or oral and maxillofacial surgery with tracheal intubation facilitated by direct laryngoscopy, videolaryngoscopy, or flexible bronchoscopy were assessed for eligibility. Patients who were scheduled for other kinds of tracheal intubation (eg, primary tracheotomy or rigid bronchoscopy), had preexisting tracheal airways (eg, tracheostomy), and pregnant patients were excluded. Each anesthetic episode with tracheal intubation was considered a case. Multiple independent assessments of participants who had multiple anesthetics were allowed as we ensured that a patient was never managed by the same anesthesiologist twice. Trainees as well as attending anesthesiologists participated; their sex, qualification, and professional work experience were recorded.

### Airway Management Planning

Patients received a structured preoperative airway risk assessment in our preassessment clinic in accordance with our in-house standards, including physical examination, medical history, simplified airway risk index, Wilson score, upper lip bite test,^10,11^ and transnasal videoendoscopy, if appropriate.^14,15,16^ The interincisor gap was measured using a single-use measuring tape with an exact millimeter scale in the midline from the upper to lower teeth or gum and grouped (>3.0 cm, 3.0-2.5 cm, 2.4-2.0 cm, and <2.0 cm) thereafter.

Self-reported symptoms were systematically assessed in all patients (eMethods 1 in Supplement 1). Physicians rated difficult airway indicators such as suspected difficult ventilation via a facemask or a supraglottic device, suspected increased risk of aspiration or rapid desaturation, suspected difficult transtracheal airway, and cervical spine immobility and/or instability as reported previously (eMethods 2 in Supplement 1).^2,9,22^ Recorded findings from previous tracheal intubations^6,17,18,19,25^ were flagged, either as red (eg, previous failed or difficult videolaryngoscopy^6^ or history of awake tracheal intubation), yellow (eg, previous failed or difficult direct laryngoscopy or history of previously required videolaryngoscopy), or green flags (neither red nor yellow flag) as shown in eMethods 2 in Supplement 1.

**Figure 1.**
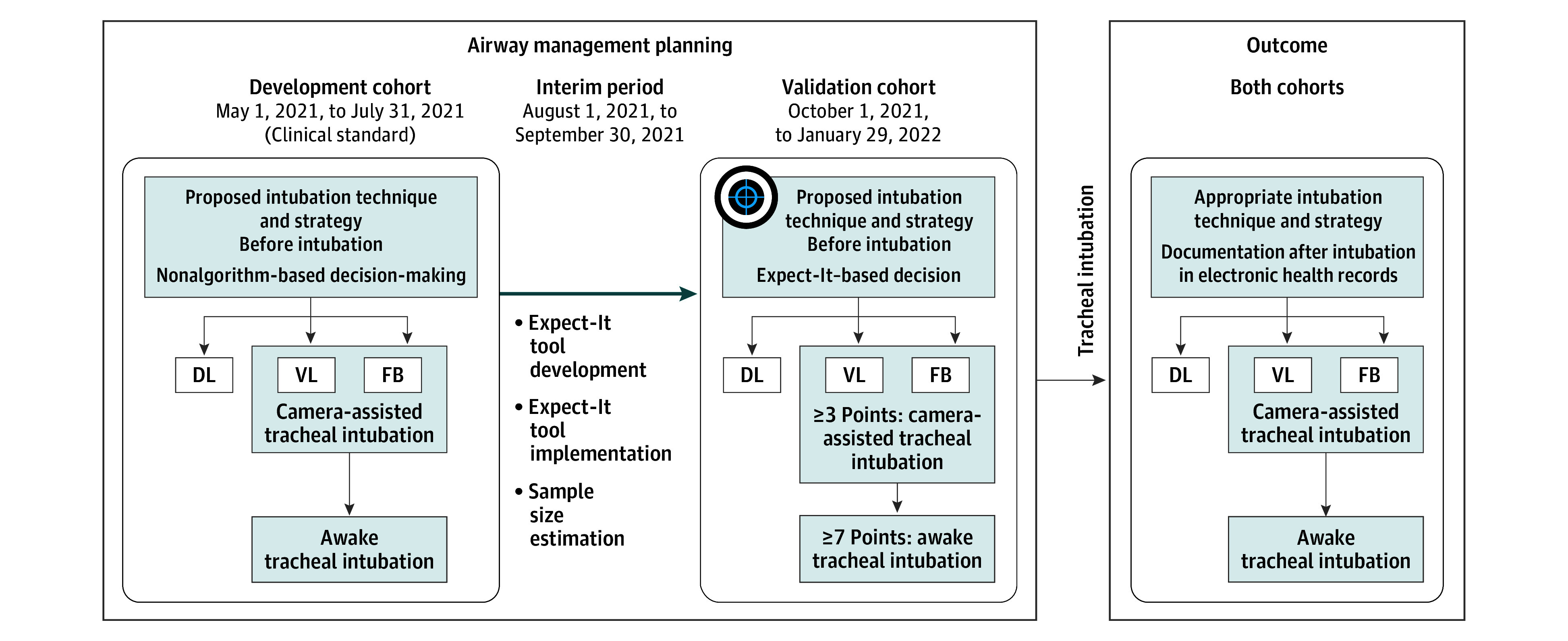
Study Design, Flow, and Timing of the Evidence-Based Algorithm for the Expected Difficult Intubation (Expect-It) Study DL indicates direct laryngoscopy; FB, flexible bronchoscopy; VL, videolaryngoscopy.

Physicians in the preassessment clinic proposed a first-line tracheal intubation technique (camera-assisted or direct laryngoscopy) and strategy (awake or asleep); this information was recorded in patients' electronic health records and used for airway management planning (Figure 1). In the development cohort, this was based on personal experience and judgment of the physicians (nonalgorithm-based decision-making, the current clinical standard). In the validation cohort, these proposals originated from the newly developed Expect-It decision-making tool (algorithm-based decision-making); here, however, anesthesiologists were encouraged to adhere to the intubation technique and strategy proposed by the decision-making tool but were free to neglect it. Camera-assisted techniques were either videolaryngoscopy or flexible bronchoscopy. Notably, direct laryngoscopy was always considered an asleep and bronchoscopy always an awake technique.

### Airway Management and Rating of the Appropriate Intubation Technique and Strategy

Macintosh (C-MAC [Karl Storz]) was considered the first-choice videolaryngoscope, and hyperangulated videolaryngoscope (C-MAC D-BLADE [Karl Storz]) was considered a rescue technique.^5,6,26^ For bronchoscopic tracheal intubation, flexible intubation videoendoscopes (5.5 × 65 or 4.0 × 65 [Karl Storz]) were used. There was no anesthesia management protocol; anesthesiologists chose drugs and dosages, how to induce anesthesia, manage the airway, and perform tracheal intubation, including the use of airway adjuncts, optimization maneuvers, and rescue techniques. Neuromuscular blocking agents were used in all anesthetized patients, and adequate neuromuscular blockade was verified by train-of-four measurements.

Directly after tracheal intubation, anesthesiologists rated which tracheal intubation technique and strategy would have been most appropriate in their individual patient. According to our in-house standard, this information was routinely recorded in patients’ electronic health records (Integrated Care Manager Anesthesia, version 13.01 [Draeger Medical]) in the form of a recommendation for future airway management planning using a structured query screen. These recordings are intended to be reused and shared for the planning of future anesthetics and in emergencies.

### Main Outcomes

Two hierarchically ordered primary outcome measures, the appropriate tracheal intubation technique (camera-assisted vs direct laryngoscopy) and strategy (awake vs asleep), were assessed.

Secondary outcome measures were overall and first attempt success rate, failed direct laryngoscopy, number of laryngoscopy and intubation attempts, tracheal introducers used, time to tracheal intubation (from device insertion to intubation of the trachea), preparation time (from topicalization to start of the endoscopy in case of awake tracheal intubation), glottic view grades (6-stage glottic view grading^5,6,19,26,27^; eMethods 2 in Supplement 1), percentage of glottic opening,^28^ classification of the difficulty of videolaryngoscopic intubation by means of the VIDIAC score,^6^ difficult face-mask ventilation, and airway-related adverse events. Objective outcome measures were assessed by an independent research assistant not involved in patient care using a standardized case report form.

### Flow and Timing

This model development and validation study had a 2-stage design (Figure 1). In the development period (May 1, 2021, to July 31, 2021), data for the development of the decision-making tool were prospectively collected. The tracheal intubation technique and strategy were proposed by the physicians based on their personal experience and judgment (nonalgorithm-based decision-making). Within an interim period (August 1 to September 30, 2021), the decision-making tool was clinically implemented (pocket cards, lectures, education, and on-site training), and the required sample size for the validation period was calculated (eMethods 3 in Supplement 1). In the validation period (October 1, 2021, to January 29, 2022), data for the validation of the decision-making tool were prospectively collected until sample size requirements were fulfilled.

### Selection of Eligible Covariables

Potentially eligible airway-related risk factors were identified by literature review, previous studies,^2,6,9,10,11,14,15,19,20,22,29,30,31,32^ and clinical considerations; the risk factors were grouped into 4 domains: (1) recordings from previous tracheal intubation difficulties^6,17,18,19,25^; (2) physical airway examination^10,11^; (3) physician’s ratings of difficult airway indicators^2,5,9,22^; and (4) suspected or verified pharyngolaryngeal lesions determined by history, self-reported symptoms, or transnasal videoendoscopy.^14,15^ Self-reported symptoms were assessed by a structured questionnaire, and eligible variables were systematically preselected from this set of symptom variables (eMethods 1 in Supplement 1). Least absolute shrinkage selector operator (LASSO) regression was used for variable selection in domains 2 to 4 (eMethods 2 in Supplement 1).^33,34^

### Regression Models

Two different regression analyses were used to obtain the Expect-It score: one to predict the appropriate tracheal intubation technique and the other to predict the appropriate strategy. For both of these outcomes, first, LASSO regression was applied to select potentially predictive variables from the large set of collected variables (eMethods 2 in Supplement 1).^33,34^ Variables assigned with a nonzero β coefficient by any of the 2 LASSO regressions were included in the multivariable logistic regression models. Complete case analysis was applied. Covariables were categorized if reasonable thresholds were identified in the LASSO regression. Variables from domain 1 were always included. Finally, scores originating from both multivariable logistic regression models were combined to create the decision-making tool.

### Score Development

β coefficients from both models were rounded to integer numbers to build the Expect-It score. To determine 1 unified point value for each covariable, β coefficients from both models were respected using predefined rules. In general, rounded β coefficients from the camera-assisted intubation model were preferred; only in domain 4, β coefficients from the awake tracheal intubation model were preferred, as there is growing evidence that pharyngolaryngeal lesions have important implications for decision-making for awake tracheal intubation.^14,15,16^

The Youden index was used to assess optimal decision thresholds. We further used the positive and negative predictive value from a utility-based perspective to determine clinically relevant thresholds, likely prompting clinical decision-making.^35^ As the decision-making tool is intended to be a diagnostic tool, we considered that it should demonstrate a positive predictive value of at least 0.5.

### Validation Methods and Performance Measures

The generalizability and performance of the decision-making tool were assessed based on data from the development and validation cohort. The area under the receiver operating characteristics curve (AUC) of the final Expect-It score to predict appropriate camera-assisted and awake tracheal intubation were calculated (R software packages ROCR and pROC [R Project for Statistical Computing]) in the development cohort, and calibration belts^36^ were plotted (R software package givitiR). The calibration of a prediction model refers to testing the agreement between the predicted and actual observed event probability.^36^

In the validation cohort, sensitivity and specificity of the decision-making tool were calculated. Both were considered coprimary end points, combined through the intersection-union test.^37^ The clinical standard, which was gathered in the development cohort, was used as a comparator. We required that superiority for sensitivity and noninferiority for specificity were demonstrated.^37,38,39^ Due to the multidimensional study design, we used a recognized hierarchical 2-step approach for testing of the primary outcomes, appropriate awake and camera-assisted tracheal intubation (hierarchically successive end point).^37,38,39,40,41,42,43^ We calculated the 2-sided 95% CI for sensitivity and specificity of the decision-making tool and compared it with the minimal sensitivity/specificity of the clinical standard; noninferiority was assumed if the lower bound of the CI was larger than the minimal specificity minus the noninferiority margin. A noninferiority margin of 5% was considered clinically relevant. Due to this hierarchical test procedure, the α adjusted for multiplicity remains the nominal significance α.^40,41,44^ The 2-sided significance level α is .05.

### Descriptive Statistics

Sample characteristics are given as frequencies and percentage values, means and SDs, or medians and IQRs, whichever is appropriate. We report odds ratios (ORs) with 95% CIs. Statistical analysis was performed using SPSS software 27 (IBM Inc) and R software, version 4.0.2 (R Project for Statistical Computing). The data were analyzed between August 2021 (first stage) and December 2023.

## Results

From 1201 patients undergoing head and neck surgery during the study period (mean [SD] age, 50.3 [19.0] years; 695 [58%] male), 1282 anesthetic cases were included in the analysis: 602 in the development and 680 in the validation cohort (eFigure 1 in Supplement 1). Demographic and baseline clinical characteristics, such as preconditions and surgical procedures, were balanced in both cohorts (eTable 1 in Supplement 1). Overall, 43 patients (3%) had a limited neck motion of less than 70°, 179 (14%) had a positive upper lip bite test result, 77 (6%) had a history of neck radiation therapy, and 83 (7%) had expanding pharyngolaryngeal lesions. Preoperative transnasal videoendoscopy was performed in 561 patients (44%). A total of 120 anesthesiologists participated (78 trainees [65%], 42 attendings [35%]; 56 female [46.7%], 64 male [53.3%]) with a median (IQR) professional work experience of 39 (5-78) months.

### Development of the Decision-Making Tool

Two different multivariable logistic regression models were fitted for both primary outcomes separately (Table 1) based on the subsets of covariables that were preselected by LASSO regression.^6,33,34^ Scores originating from both models were combined to give the decision-making tool. For score development, β coefficients were rounded to integer numbers, and β coefficients from both models were respected using the designated rules outlined in the material section. As β coefficients for a mouth opening less than 2.0 cm had a wide 95% CI and differed between both models, it was decided to assign an additional 5 points and that decision-making for awake tracheal intubation should be individualized in these patients. As the β coefficient for a relevantly restricted glottic view^14,15^ was high with a wide 95% CI, it was recommended in this study that these individuals should directly be considered for awake tracheal intubation.

**Table 1.** Two Multivariable Logistic Regression Models to Predict Appropriate Tracheal Intubation Technique and Strategy^a^

Characteristic	β Coefficient (95% CI)	
	Appropriate camera-assisted intubation	Appropriate awake intubation
Domain 1: previous tracheal intubations^b^		
Green flag	−0.27 (−0.95 to 0.36)	0.82 (−1.08 to 2.81)
Yellow flag	1.20 (−0.23 to 2.55)	1.44 (−0.99 to 3.88)
Red flag	3.49 (2.25 to 5.03)	2.39 (0.56 to 4.43)
Domain 2: physical airway examination^c^		
Mouth opening		
>3 cm	[Reference]	[Reference]
3.0-2.5 cm	1.03 (−0.16 to 2.21)	1.21 (−0.84 to 3.19)
2.4-2.0 cm	2.63 (1.14 to 4.34)	1.25 (−0.96 to 3.42)
<2.0 cm	15.50 (−38.00 to 425.01)	2.25 (0.03 to 4.73)
Mallampati class 4	0.74 (−0.14 to 1.57)	−0.11 (−1.85 to 1.53)
Cannot bite upper lip	0.99 (0.29 to 1.68)	NI^d^
Retrognathia	0.96 (0.35 to 1.56)	NI^d^
Neck movement		
>90°	[Reference]	NI^d^
70°-90°	0.44 (−0.17 to 1.08)	NI^d^
<70°	2.51 (1.48 to 3.55)	NI^d^
Domain 3: physician’s ratings of difficult airway indicators^c^		
Suspected difficult supraglottic airway device	1.15 (0.25 to 2.04)	3.00 (1.34 to 5.06)
Domain 4: pharyngolaryngeal lesions^c^		
Expanding lesions	0.77 (−0.40 to 1.84)	0.67 (−1.07 to 2.35)
History of neck radiotherapy	0.45 (−0.81 to 1.63)	0.98 (−0.53 to 2.47)
Dysphagia	NI^d^	1.09 (−0.32 to 2.52)
Transnasal videoendoscopy^e^		
No pharyngolaryngeal lesions	NI^d^	0.09 (−1.60 to 1.71)
Vestibular fold lesion	NI^d^	4.70 (1.23 to 7.95)
Relevantly restricted glottic view	NI^d^	22.70 (−154.23 to 1074.21)

Abbreviation: NI, not included.

^a^ Models were based on 602 cases from the development cohort. Due to 3 missing values for mouth openings, only 599 cases were included in the complete case analysis. The models predicted the appropriate technique, either camera-assisted or direct laryngoscopy, and the correct strategy, either awake or asleep tracheal intubation.

^b^ The presence of red flags (eg, previous failed or difficult videolaryngoscopy^6^ or history of awake tracheal intubation), yellow flags (eg, previous failed or difficult direct laryngoscopy or history of previously required videolaryngoscopy), or green flags (neither red nor yellow flag) was always included in both models (eMethods 2 in Supplement 1).

^c^ Only selected potentially predictive covariables (least absolute shrinkage selector operator [LASSO]; eMethods 2 in Supplement 1) were included.

^d^ Shrunk to 0 in the LASSO regression.

^e^ Recategorized after LASSO regression: relevantly restricted glottic view comprises relevant view restrictions less than 50% and 50% or more of the glottis area.

**Figure 2.**
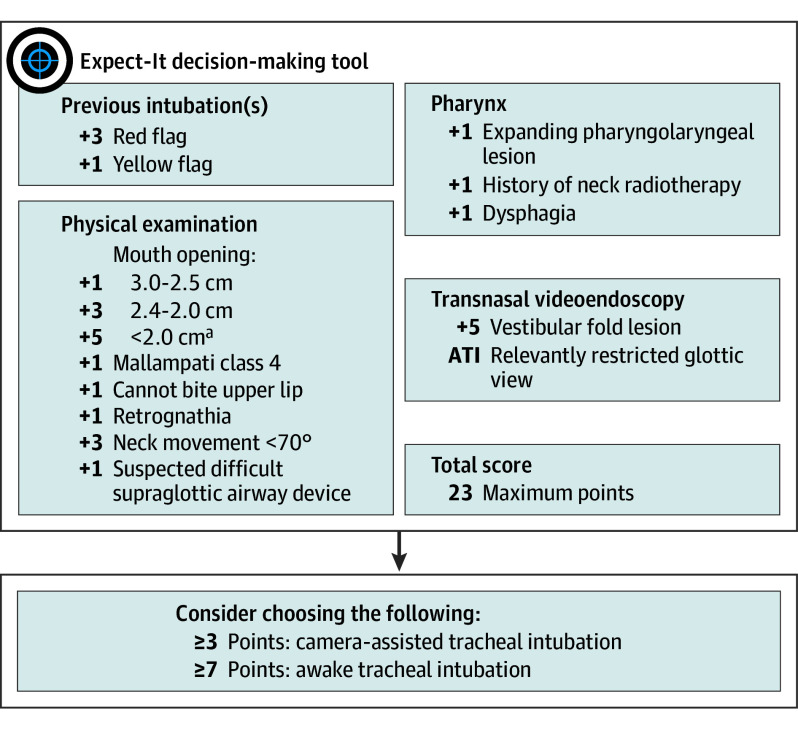
The Evidence-Based Algorithm for the Expected Difficult Intubation (Expect-It) Decision-Making Tool A previous failed or difficult videolaryngoscopy^6^ (eg, a Videolaryngoscopic Intubation and Difficult Airway Classification [VIDIAC] score ≥3) or history of awake tracheal intubation was considered a red flag; a previous failed or difficult direct laryngoscopy, a VIDIAC score of 2, or history of previously required videolaryngoscopy was considered a yellow flag (eMethods 2 in Supplement 1). ATI indicates awake tracheal intubation. ^a^Individualized decision for or against awake tracheal intubation based on the total score and clinical considerations (eg, pain-related small mouth opening).

Figure 2 illustrates the final Expect-It decision-making tool. The overall Expect-It score ranges between 0 and 23 points. A score of 3 or more points achieved the highest Youden index for both primary outcome measures (0.59 for camera-assisted and 0.85 for awake tracheal intubation, respectively); further, a score of 7 points achieved a positive predictive value of greater than 0.5 for the prediction of appropriate awake tracheal intubation (eTable 2 in Supplement 1).

### Validation Testing and Performance Measures

**Figure 3.**
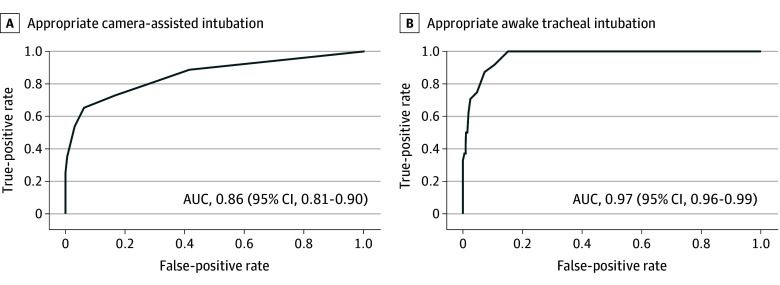
Receiver Operating Characteristic Curves of the Tool’s Aptitude Predicting Appropriate Awake and Camera-Assisted Tracheal Intubation in the Development Cohort AUC indicates area under the receiver operating characteristic curve.

The distribution of the Expect-It score in the validation cohort is shown in eFigure 2 in Supplement 1. The decision-making tool achieved good discrimination to predict appropriate camera-assisted tracheal intubation (AUC, 0.86 [95% CI, 0.81-0.90]) and awake tracheal intubation (AUC, 0.97 [95% CI, 0.96-0.99]) (Figure 3) and calibration belts showed good agreement between the observed and predicted probabilities within the entire range of probabilities, and never crossed the diagonal bisector line, indicating good model calibration (eFigure 3 in Supplement 1).^36^

A total of 660 of 680 airway operators (97%) adhered to the intubation technique and strategy proposed by the decision-making tool in the validation cohort. Misclassifications with the decision-making tool were rare; most were about the technique (direct laryngoscopy instead of camera-assisted intubation in 15 cases and camera-assisted instead of direct laryngoscopy in another 15 cases) and rarely about the strategy (awake instead of asleep tracheal intubation in 1 patient and asleep instead of awake in 1 other patient).

For both primary outcome measures, specificity of the decision-making tool in the validation cohort vs the clinical standard obtained in the development cohort was noninferior (camera-assisted tracheal intubation: 97% [95% CI, 96%-98%] vs 96% [95% CI, 93%-97%], respectively; awake tracheal intubation: 100% [95% CI, 99%-100%] vs 98% [95% CI, 97%-99%], respectively) and sensitivity superior (camera-assisted tracheal intubation: 88% [95% CI, 81%-93%] vs 35% [95% CI, 27%-44%], respectively; awake tracheal intubation: 97% [95% CI, 81%-100%] vs 29% [95% CI, 15%-50%], respectively) (eTable 3 in Supplement 1).

### Secondary Outcomes

After clinical implementation of the decision-making tool, videolaryngoscopy was more frequently used first line (development vs validation cohort, 53 [9%] vs 90 [13%] cases, respectively; OR, 1.58 [95% CI, 1.10-2.26]) but less often required to rescue failed direct laryngoscopy (development vs validation cohort, 45 [8%] vs 10 [2%] cases, respectively; OR, 0.18 [95% CI, 0.09-0.37]) and the first attempt success rate increased (development vs validation cohort, 437 [73%] vs 557 [82%] cases, respectively; OR, 1.72 [95% CI, 1.32-2.22]). Airway-related adverse events decreased (development vs validation cohort, 68 [11%] vs 31 [5%] cases, respectively; OR, 0.37 [95% CI, 0.24-0.58)]) after implementation (Table 2). Further secondary outcomes are given in Table 2.

**Table 2.** Secondary Study Outcomes

Characteristic	No. (%)		OR (95% CI)
	Development cohort (n = 602)	Validation cohort (n = 680)	
Tracheal intubation technique and strategy			
Camera-assisted tracheal intubation			
Videolaryngoscopy	53 (9)	90 (13)	1.58 (1.10-2.26)
Bronchoscopy	24 (4)	29 (4)	1.07 (0.62-1.86)
Direct laryngoscopy	525 (87)	561 (83)	0.69 (0.51-0.94)
Awake tracheal intubation	24 (4)	29 (4)	1.07 (0.62-1.86)
Secondary outcome parameter			
Overall successful first-line technique	552 (92)	667 (98)	4.55 (2.50-8.33)
Failed direct laryngoscopy (conversion to videolaryngoscopy)	45 (8)	10 (2)	0.18 (0.09-0.37)
First attempt success^a^	437 (73)	557 (82)	1.72 (1.32-2.22)
Laryngoscopy attempts			
1	473 (79)	587 (86)	1.72 (1.28-2.32)
2	88 (15)	71 (10)	0.68 (0.49-0.95)
≥3	41 (7)	22 (3)	0.46 (0.27-0.78)
Intubation attempts			
1	516 (86)	593 (87)	1.14 (0.83-1.56)
2	60 (10)	71 (10)	1.05 (0.74-1.52)
≥3	26 (4)	16 (2)	0.53 (0.28-1.00)
Tracheal introducers used	19 (3)	9 (1)	0.41 (0.18-0.92)
Time to tracheal intubation, median (IQR), s^b^	43 (23-107)	41 (23-97)	NA
Preparation time (for awake tracheal intubation only; n = 53), median (IQR), s^c^	842 (573-1418)	925 (638-1292)	NA
Glottic view grades^d^^,^^e^			
Vocal cords clearly visible	453 (75)	592 (87)	2.22 (1.67-2.94)
Vocal cords only just visible	104 (17)	62 (9)	0.48 (0.34-0.67)
Vocal cords not visible	45 (8)	25 (4)	0.47 (0.29-0.78)
Percentage of glottic opening, median (IQR), %^e^	85 (50-100)	90 (75-100)	NA
VIDIAC score^f^			
2 (Hard videolaryngoscopic intubation)	13 (2)	11 (2)	0.75 (0.33-1.67)
≥3 (Severe videolaryngoscopic intubation)	16 (3)	13 (2)	0.16 (0.05-0.56)
Difficult face mask ventilation	47 (8)	47 (7)	0.88 (0.57-1.33)
≥1 Airway-related adverse event(s)	68 (11)	31 (5)	0.37 (0.24-0.58)
Failed first-line technique	50 (8)	13 (2)	NA
Hypoxia (SpO_2_ <90%)	12 (2)	11 (2)	NA
Aspiration	1 (0.2)	1 (0.1)	NA
Glottic swelling^g^	2 (0.3)	1 (0.1)	NA
Laryngospasm	1 (0.2)	0	NA
Dental or soft tissue injury	6 (1)	1 (0.1)	NA
Oral bleeding	14 (2)	5 (0.7)	NA

Abbreviations: NA, not applicable; OR, odds ratio; SpO_2_, oxygen saturation of pulse; VIDIAC, Videolaryngoscopic Intubation and Difficult Airway Classification.

^a^ One attempt at laryngoscopy and intubation.

^b^ From device insertion to intubation of the trachea.

^c^ From topicalization to start of the endoscopy.

^d^ Rated using a recognized 6-stage grading system^5,6,19,26,27^ (vocal cords completely visible, part of the cords visible, posterior cords only just visible, arytenoids but not cords visible, epiglottis but no glottis visible, laryngeal structures not visible; eMethods 2 in Supplement 1).

^e^ Only for the first-line technique.

^f^ For first-line or rescue videolaryngoscopy.

^g^ With corticosteroids given.

## Discussion

This prospective study on model development and validation used a bottom-up data-driven approach to develop a decision-making tool for airway management planning in patients undergoing head and neck surgery; hence, it relied on cross-sectional clinical data. The Expect-It study was a pilot to use electronic health care data from multiple sources to support decision-making during airway-management planning and is a precursor for the development of intelligent algorithms. The Expect-It decision-making tool demonstrated high discrimination for the prediction of appropriate camera-assisted and awake tracheal intubation.

Expect-It provides physicians with a concrete, reliable decision support for airway management planning and avoids relevant diagnostic gaps by taking structured data from 4 key domains into account: recordings from previous tracheal intubation difficulties,^6,17,18,19,25^ physical examination,^10,11^ physician’s rating of difficult airway indicators,^2,9,22^ and suspected or verified pharyngolaryngeal lesions determined by history, self-reported symptoms, and/or preoperative transnasal videoendoscopy.^14,15,16^ Clinicians can use this patient-specific information to calculate the Expect-It score; a score of 3 or higher supports using a camera-assisted technique, while a score of 7 or higher supports awake tracheal intubation.

Our findings suggest that the decision-making tool presented herein could increase the sensible and targeted use of camera-assisted tracheal intubation and awake tracheal intubation, which might currently be underused.^1^ After the clinical implementation of the decision-making tool in our department, the first attempt success and overall success rates increased, while failed direct laryngoscopy was rarely observed. Hence, patients underwent the optimal intubation techniques more often from the beginning. Beyond these improved clinician-centered outcomes, patient-centered outcomes, especially airway-related adverse events, also decreased.

Do we have to rethink preoperative airway assessment? Would decision-making tools not be more appropriate than airway risk assessment tests? Decision-making tools have been increasingly promoted for anesthesia planning,^2,9,20,22,45^ and Expect-It is a prospective, quantifiable tool with confirmed accuracy. Reliable identification of patients at high risk for complications and proper use of airway management techniques might decrease the risk of adverse airway events without indiscriminately overutilizing health care resources.

Current randomized studies^7,46^ and a recent Cochrane review^3^ reported that videolaryngoscopy improved success rates and patient safety in adults compared with direct laryngoscopy. Our data indicate that a more personalized use of camera-assisted and awake tracheal intubation might be associated with improved success rates and outcomes. The Expect-It concept offers a targeted and sensible approach, as distinct cutoffs for the indication of camera-assisted and awake tracheal intubation are proposed and possible limitations for asleep intubation are outlined.

Our data show that severely restricted mouth opening and expanding pharyngolaryngeal lesions were the strongest indication criteria for awake tracheal intubation. An interincisor gap of less than 3.0 cm has been considered a risk factor for difficult videolaryngoscopic intubation in previous studies.^47,48,49,50^ A recent study found that patients with mouth openings less than 3.0 cm could be managed safely with hyperangulated videolaryngoscopy if given limitations were respected.^51^ Our data suggest that videolaryngoscopic intubation must be critically appraised in individuals with a mouth opening less than 2.5 cm.

### Limitations

As the Expect-It study was conducted at a single center in adults undergoing head and neck surgery, and standards, equipment, and populations may differ between institutions and regions, findings should not be generalized or extrapolated to other institutions or cohorts. Studies with temporal cohorts are inherently susceptible to bias; to reduce bias, the time lag between cohorts was minimized.^52^ However, baseline characteristics between cohorts are balanced. Additional factors such as syndromes, comorbidities, anxiousness, or patient nonadherence might not be reflected; hence, the decision-making tool can only be supportive; the final individualized decision must be made dependent on the context by a skilled physician. Our study was conducted in a representative cross-sectional study cohort. Further external validation in other settings and populations, for example, in selected cohorts at high risk for complications, could reinforce our findings.

## Conclusions

In this study, the Expect-It decision-making tool to provide a rational decision-making support for camera-assisted and awake tracheal intubation was developed and validated. The decision-making tool integrates data and diagnostics from multiple sources, such as structured reports from previous intubation difficulties, transnasal videoendoscopy, and symptoms for pharyngolaryngeal lesions, and it demonstrated excellent diagnostic performance. It may serve as a precursor for intelligent algorithms. After clinical implementation of the decision-making tool, camera-assisted and awake tracheal intubations were used in a more targeted manner, and airway-related adverse events decreased.

## Notes

Supplement 1. **eMethods 1.** Patient’s self-reported symptoms—selecting potential predictors **eMethods 2.** Specification and selection of eligible covariables **eMethods 3.** Sample size analysis **eFigure 1.** Enrollment **eTable 1.** Characteristics of the study cohorts **eTable 2** Optimal decision thresholds of the Expect-It score **eFigure 2.** Histogram showing the distribution of the Expect-It score in the validation cohort (n = 680). **eFigure 3.** Calibration belts **eTable 3.** Accuracy of preoperative decision-making
eReferences

Supplement 2.
Data Sharing Statement
