# Supplementary material for: Decision-Making Tool for Planning Camera-Assisted and Awake Intubation in Head and Neck Surgery
Source: JAMA Otolaryngol Head Neck Surg. 2025 May 1;151(6):585–94. doi: 10.1001/jamaoto.2025.0538 (PMC12046521; doi:10.1001/jamaoto.2025.0538)
Supplement: Supplement 1. — eMethods 1. Patient’s self-reported symptoms—selecting potential predictors eMethods 2. Specification and selection of eligible covariables eMethods 3. Sample size analysis eFigure 1. Enrollment eTable 1. Characteristics of the study cohorts eTable 2 Optimal decision thresholds of the Expect-It score eFigure 2. Histogram showing the distribution of the Expect-It score in the validation cohort (n = 680). eFigure 3. Calibration belts eTable 3. Accuracy of preoperative decision-making eReferences [file jamaotolaryngolheadnecksurg-e250538-s001.pdf]

Supplemental Online Content

Popal Z, Sieg H, Müller-Wiegand L, et al. Decision-making tool for planning camera-assisted and awake intubation in head and neck surgery. *JAMA Otolaryngol Head Neck Surg.* Published online May 1, 2025.
doi:10.1001/jamaoto.2025.0538

eMethods 1. Patient's self-reported symptoms—selecting potential predictors

eMethods 2. Specification and selection of eligible covariables

eMethods 3. Sample size analysis

eFigure 1. Enrollment

eTable 1. Characteristics of the study cohorts

eTable 2 Optimal decision thresholds of the Expect-It score

eFigure 2. Histogram showing the distribution of the Expect-It score in the validation cohort (n = 680).

eFigure 3. Calibration belts

eTable 3. Accuracy of preoperative decision-making

eReferences

This supplemental material has been provided by the authors to give readers additional information about their work.

© 2025 Popal C et al. *JAMA Otolaryngology–Head & Neck Surgery*.

eMethods1. Patient's self-reported symptoms - selecting potential predictors

Prior to the main analysis thirteen self-reported symptoms were systematically assessed using a questionnaire in all patients. Random forest analysis was used to select potential predictors for the appropriate tracheal intubation technique (camera-assisted versus direct laryngoscopy) and strategy (awake versus asleep tracheal intubation). Random forest analysis was based on 500 decision trees. Gini impurity was calculated to quantify the importance of each variable to predict the appropriate tracheal intubation technique and strategy. Only the three variables with the highest importance in the random forest analysis were considered potentially relevant. Only self-reported symptom variables that were selected for the appropriate intubation technique and strategy were considered eligible and used for further analysis within *domain 4*.

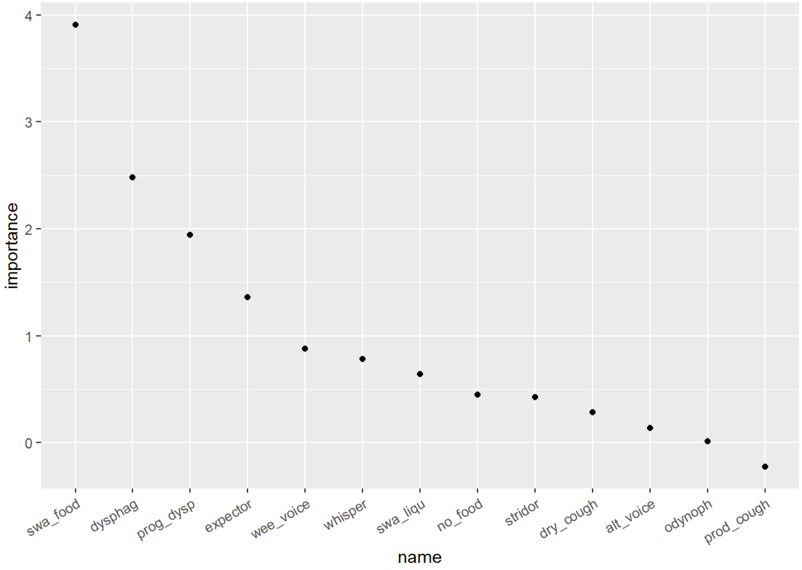

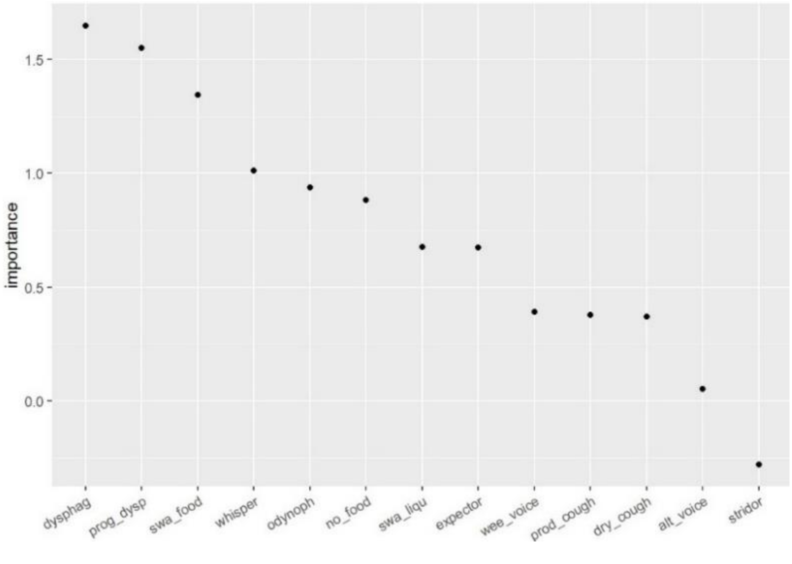

Random forest analysis for selecting potentially eligible self-reported symptom variables for the prediction of the appropriate tracheal intubation technique (above) and strategy (below); y-axis: variable importance; x-axis: thirteen self-reported symptom variables; dysphag, dysphagia (yes/no); prog_dysp, progressive dysphonia in the last 3 months (yes/no) swa_food, difficulties swallowing solid food (yes/no); whisper, whispering or aphonia (yes/no); odynoph, odynophagia (yes/no); no_food, inability of food intake (yes/no); swa_liqu, difficulties swallowing liquids (yes/no); expector, impaired expectoration (yes/no); wee_voice, week voice (yes/no); prod_cough, productive cough (yes/no); dry_cough, dry cough (yes/no); alt_voice, altered voice (yes/no); stridor, inspiratory stridor (yes/no)

© 2025 Popal C et al. *JAMA Otolaryngology–Head & Neck Surgery*.

eMethods2. Specification and selection of eligible covariables

Airway-related risk factors for the prediction of both primary outcomes, appropriate tracheal intubation technique (camera-assisted versus direct laryngoscopy) and strategy (awake versus asleep), were identified by literature research, previous studies, and clinical considerations. Factors were grouped into FOUR DOMAINS:

- ***Domain 1:*** Previous tracheal intubations (history of difficult laryngoscopy/ difficult tracheal intubation)
- ***Domain 2:*** Physical airway examination (findings from preoperative airway risk assessment tests)
- ***Domain 3:*** Physician's ratings of difficult airway indicators (structured assessment of indicators gathered from established pre-airway management decision-making tools (modified after[1-3])
- ***Domain 4:*** Pharyngolaryngeal lesions: Verified or suspected lesions based on medical history, patient's self-reported symptoms (only preselected potentially eligible self-reported symptom variables; eMethods1) and preoperative transnasal videoendoscopy[4,5]

<u>Specification of airway-related risk factors</u>

<u>DOMAINE 1: Previous tracheal intubations; definition of flags</u>

**Flags for previous difficult intubations**	**Definition criteria (at least one)**
**Red flag**<br>(high risk)	**Videolaryngoscopy:** Reported impaired videolaryngoscopic glottic view[6-8] (see below); VIDIAC score ≥3[7]; previously failed videolaryngoscopy (conversion to another intubation technique such as bronchoscopy or abandoned intubation)<br>**Direct laryngoscopy:** Reported: Laryngeal structures not visible with direct laryngoscopy[6,7,9]<br>**Airway alert:** Documented difficult videolaryngoscopy alert or previous awake tracheal intubation (e.g. anaesthesia chart or alert card) or written recommendation for awake tracheal intubation for future anesthetics
**Yellow flag**<br>(intermediate risk)	**Videolaryngoscopy:** Reported restricted videolaryngoscopic glottic view[6-8,10] (see below); VIDIAC score 2[7]; previously required videolaryngoscopy<br>**Direct laryngoscopy:** Reported impaired direct glottic view[6-8]; failed direct laryngoscopy (conversion to another intubation technique such as videolaryngoscopy)<br>**Airway alert:** Documented difficult direct laryngoscopy alert (e.g. anaesthesia chart or alert card) or written recommendation given for videolaryngoscopy for future anesthetics
**Green flag**<br>(low risk)	Absence of a yellow or red flag

The history of difficult tracheal intubation or difficult laryngoscopy relied on existing reports from previous tracheal intubations. Variables were grouped in three risk categories (green, yellow or red flag) by clinical considerations and literature review.[6-13] VIDIAC score: videolaryngoscopic intubation and difficult airway classification score[7]

© 2025 Popal C et al. *JAMA Otolaryngology–Head & Neck Surgery*.

Six-staged grading of the glottic view

Six-staged grading of the glottic view gathered during (video-) laryngoscopy as previously reported[6-8,12,14,15] based on recognized landmarks[9,10,13]

Vocal cords	Visible landmarks	Glottic view	
Clearly visible	Vocal cords completely visible	Good	
	Part of the cords visible		
Only just visible	Posterior cords only just visible	Restricted	
Not visible	Arytenoids but not cords visible		Impaired
	Epiglottis but no glottis visible		
	Laryngeal structures not visible		

DOMAIN 2: Physical airway examinations; preoperative airway risk assessment tests

- Mouth opening (cm): The inter-incisor gap was measured using a single-use measuring tape with an exact millimeter scale in the midline from the upper to lower teeth or gum
- Mallampati (grade I-IV) as defined previously[16]
- Upper lip bit test (class I: lower incisors extend beyond the vermilion border of the upper lip; class 2: lower incisors bite the lip but cannot extend above the vermilion border and class 3 lower incisors cannot bite the upper lip at all) as defined previously [17]
- Lower teeth don't meet upper teeth (yes/ no) as defined previously[18]
- Retrognathia (no, moderate, severe) as defined previously[18]
- Neck movement (degree) measured and categorized (<70°, 70-79°, 80-89°, 90°, >90°)
- Thyromental distance (mm) measured during maximal neck extension
- Prominent incisors (no/moderate/severe) as defined previously[18]
- Age (years)
- Weight (kg)
- Sex (male/female)

DOMAIN 3: Physician's subjective ratings of difficult airway indicators

**Physician's subjective ratings of difficult airway indicator inspired by previously reported pre-airway management decision-making tools[1-3]**
• Suspected difficult facemask ventilation (yes/no)
• Suspected difficult ventilation with a supraglottic device (yes/no)
• Suspected significantly increased risk of aspiration with indicated rapid sequence intubation (yes/no)
• Suspected increased risk for rapid desaturation (yes/no)
• Suspected difficult transtracheal airway (yes/no)
• Cervical spine immobility/ instability (yes/no)

DOMAIN 4: Pharyngolaryngeal lesions

Suspected or verified pharyngolaryngeal lesions determined by:

Medical history:

- Expanding pharyngolaryngeal lesions (yes/no): history of known expanding pharyngolaryngeal lesions
- History of neck radiotherapy (yes/no)

Self-reported symptoms:

- Dysphagia (yes/no)*
- Difficulties swallowing solid food (yes/no)*
- Progressive dysphonia in the last 3 months (yes/no)*

*These three self-reported symptom variables were selected by random forest analysis out of 13 self-reported variables (questionnaire) (eMethods1).

Transnasal videoendoscopy findings (details are given elsewhere[4,5])

- Vestibular fold lesion (yes/no)
- Arytenoid lesion (yes/no)

© 2025 Popal C et al. *JAMA Otolaryngology–Head & Neck Surgery*.

- Epiglottis lesion (yes/no)
- Supraglottic lesion (yes/no)
- Hypopharynx lesion (yes/no)
- Multiple unilateral lesions (yes/no)
- Unrestricted view on the rima glottidis (yes/no)
- Relevant view restriction on the rima glottidis that cover less than half of the glottis cross-sectional area (yes/no)
- Relevant view restriction on the rima glottidis that cover more than half of the glottis cross-sectional area (yes/no)

Selection of airway-related risk factors for the development of the Expect-It tool

LASSO regression analysis was used in the development cohort to select potentially predictive co-variables from a set of airway-related risk factors.[19,20] Airway-related risk factors were selected if considered relevant for the prediction of the appropriate tracheal intubation technique (camera-assisted or direct laryngoscopy) and strategy (awake or asleep tracheal intubation) within each *domain 2, 3 and 4* independently. Thus, overall six LASSO regression models were calculated for this approach. The shrinkage parameter λ was obtained using 10-fold cross-validation and the largest λ within one standard error of the minimum of the mean cross-validated error was chosen (λ_{1se}) to estimate the shrunken β-coefficients. Coefficients were calculated for each airway-related risk factor for both primary outcomes. Coefficients from the best-fitting LASSO regression models, which were not shrunk to zero in the LASSO regressions, were considered relevant and used for the fitting of the final multivariable logistic regression models (Table 1 main manuscript). The variables in *domain 1* were always included in the final multivariable logistic regression models without prior selection.

**LASSO regression analysis for the selection of airway-related risk factors within *domains 2-4***			
**Tracheal intubation technique and strategy**		**Appropriate awake tracheal intubation** β-coefficient	**Appropriate camera-assisted tracheal intubation** β-coefficient
**Physical airway examination** *(DOMAIN 2)*		n=599* λ=0.009	n=599* λ=0.01
Intercept		-2.59	1.15
Mouth opening (cm)		-0.138	-0.63
Mallampati grade	I	*ST 0*	*ST 0*
	II	*ST 0*	*ST 0*
	III	*ST 0*	*ST 0*
	IV	ST 0	0.361
Upper lip bit test class	I	*ST 0*	*ST 0*
	II	*ST 0*	*ST 0*
	III	*ST 0*	0.41
Lower teeth don't meet upper teeth		*ST 0*	*ST 0*
Prominent incisors	moderate	*ST 0*	*ST 0*
	severe	*ST 0*	*ST 0*
Retrognathia	moderate	*ST 0*	0.054
	severe	*ST 0*	*ST 0*
Neck movement	<70°	*ST 0*	0.56
	70-79°	*ST 0*	*ST 0*
	80-89°	*ST 0*	*ST 0*
	90°	*ST 0*	*ST 0*
	>90°	*ST 0*	-0.168
Thyromental distance (mm)		*ST 0*	*ST 0*
Age (years)		*ST 0*	*ST 0*

© 2025 Popal C et al. *JAMA Otolaryngology–Head & Neck Surgery*.

Weight (kg)	*ST 0*	*ST 0*
Sex (male/ female)	*ST 0*	*ST 0*
**Physician's ratings of difficult airway indicators** (*DOMAIN 3*)	n=602<br>λ=0.009	n=602<br>λ=0.01
Intercept	-3.75	-1.60
Suspected difficult facemask ventilation	*ST 0*	*ST 0*
Suspected difficult ventilation with a supraglottic device	2.29	1.56
Suspected risk of aspiration	*ST 0*	*ST 0*
Suspected risk for rapid desaturation	*ST 0*	*ST 0*
Suspected difficult transtracheal airway	*ST 0*	*ST 0*
Cervical spine immobility/ instability	*ST 0*	*ST 0*
**Pharyngolaryngeal lesions** *(DOMAIN 4)*	n=602<br>λ=0.011	n=602<br>λ=0.01
Intercept	-0.88	-2.09
Expanding pharyngolaryngeal lesions	0.68	1.18
History of neck radiotherapy	1.16	1.25
Self-reported symptoms		
Dysphagia	0.84	*ST 0*
Difficulties swallowing solid food	*ST 0*	*ST 0*
Progressive dysphonia in the last 3 months	*ST 0*	*ST 0*
Transnasal videoendoscopy[4,5]		
Vestibular fold lesion	1.03	*ST 0*
Arytenoid lesion	*ST 0*	*ST 0*
Epiglottis lesion	*ST 0*	*ST 0*
Supraglottic lesion	*ST 0*	*ST 0*
Hypopharynx lesion	*ST 0*	*ST 0*
Multiple unilateral lesions	*ST 0*	*ST 0*
Unrestricted view on the rima glottidis	-2.99	*ST 0*
View restriction on the rima glottidis		
<50% of the glottis area	*ST 0*	*ST 0*
≥50% of the glottis area	*ST 0*	*ST 0*

*LASSO regression analysis was applied in each domain 2-4 independently; reported are λ-shrinkage parameter and β-coefficients; *due to 3 missing values (mouth opening) only 599 cases could be included in domain 2; ST 0: shrunk to zero*

© 2025 Popal C et al. *JAMA Otolaryngology–Head & Neck Surgery*.

eMethods 3. Sample size analysis

As our research question is novel and appropriate data for sample size estimation were unavailable, we choose a pragmatic explorative approach for the development period and decided to include all available cases within a designated study period of three months. Based on institutional case numbers and own pretrial experiences[7,21,22] we expected a case number of approximately n=600 anesthetics in eligible patients that would consent to participate within this three months period (development cohort). In general, based on previous study findings[7,21,22] and in-house data, the prevalence of camera-assisted intubation is 11% and 2% for awake tracheal intubation.[23-26] As the risk for difficult laryngoscopy and intubation is much higher in head and neck surgery[4,7,27,28] we expected a 1.5 to 2-times higher rate for camera-assisted and awake tracheal intubation in our study cohort.

We further decided to conduct the study in two phases (development and validation period) with one analysis in between. The aim of that analysis was to develop the Expect-It tool and to perform a sample size estimation for the validation period. The study was then continued until all requirements of this sample size analysis were fulfilled.

<u>Key assumptions:</u> In our study sensitivity and specificity were considered co-primary end points. We analyzed the ability to predict two primary outcome measures, (i) the appropriate intubation technique (camera-assisted versus direct laryngoscopy) and (ii) the appropriate intubation strategy (awake or asleep) using a recognized two-step approach for testing[29-32] and determined that superiority for sensitivity and noninferiority for specificity had to be demonstrated for both primary outcomes.[33-35] We determined that the study will only be completed if all sample sizes requirement for both primary outcome measures would be reached.

<u>Sample size analysis:</u>

Our planned interim analysis based on the data of the development cohort (n=602) revealed that 20% of the cases were finally successfully managed by camera-assisted intubation and 4% with awake tracheal intubation (ATI). The sensitivity values for sample size approximation for the appropriate tracheal intubation technique (35% for camera-assisted intubation) and strategy (29% for awake tracheal intubation) as well as the specificity values (96% and 98%, respectively) originate from the data of the development cohort that were analyzed within the planned interim analysis. A 13% improvement of the sensitivity to predict appropriate camera-assisted tracheal intubation and a 26% improvement of the sensitivity to predict appropriate awake tracheal intubation were considered clinically relevant as this might avoid relevant over- and undertreatment and was therefore considered the smallest effect worthwhile detecting in this study.[36]

Based on these findings the required sample size for the confirmation of the diagnostic accuracy (validation cohort) was calculated (September 10, 2021) and submitted to the Ethics Committee of the Medical Association of Hamburg (amendment 2021-10459_2-BO-ff, December 3, 2021).

<u>Sample size calculation for camera-assisted intubation</u>

With a data sample of 556 cases and a primary outcome prevalence of 20% for camera-assisted intubations we will be able to show superiority of the experimental test regarding a sensitivity of 48% compared with a minimal sensitivity of 35% (interim analysis) for the current standard and noninferiority regarding the specificity compared with a minimal specificity of 96% of the current standard with a noninferiority margin of 5%. The two-sided significance level is 5% for each end point and the overall power is 80%. Giving these assumptions, at least 111 camera-assisted and at least 441 direct laryngoscopic intubations must be included.

<u>Sample size calculation for awake tracheal intubation</u>

With a data sample of 600 cases and a primary outcome prevalence of 4% for ATI we will be able to show superiority of the experimental test regarding a sensitivity of 56% compared with a minimal sensitivity of 29% (interim analysis) for the current standard and noninferiority regarding the specificity of the experimental test compared with a minimal specificity of 99% for the current standard with a noninferiority margin of 5%. The two-sided significance level is 5% for each end point and the overall power is 80%. Giving these assumptions, at least 24 awake and at least 276 asleep tracheal intubations must be included.

© 2025 Popal C et al. *JAMA Otolaryngology–Head & Neck Surgery*.

eFigure 1. Enrollment

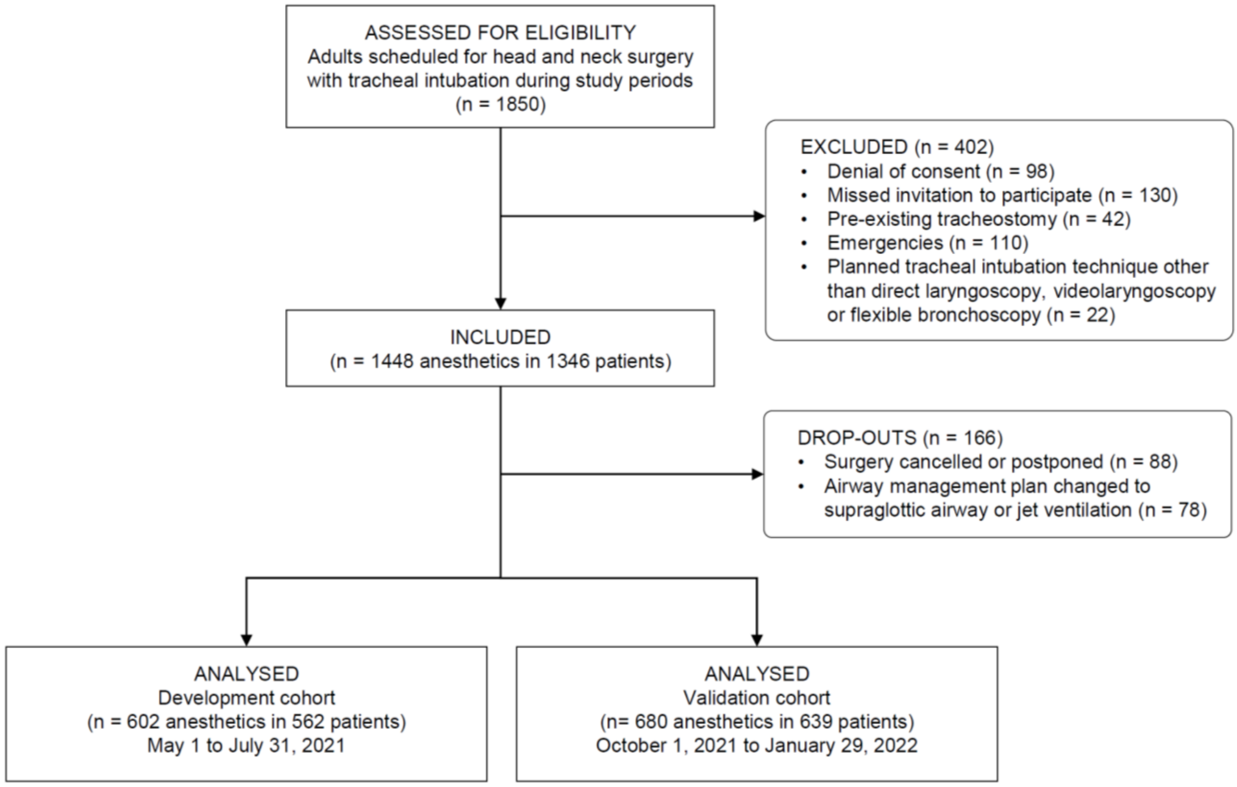

© 2025 Popal C et al. *JAMA Otolaryngology–Head & Neck Surgery*.

eTable1. **Characteristics of the study cohorts**

Characteristic	Development cohort (n=602)	Validation cohort (n=680)	Overall (n=1282)
Age; mean (SD), y	51.1 (19.2)	50.7 (18.8)	50.9 (19.0)
Sex; male, No. (%)	349 (58)	390 (57)	740 (58)
Weight; mean (SD), kg	77.4 (18.1)	78.4 (18.4)	77.9 (18.2)
ASA, No. (%)			
1	156 (26)	156 (23)	312 (24)
2	304 (51)	349 (51)	653 (51)
3	138 (23)	172 (25)	310 (24)
4	4 (0.7)	3 (0.4)	7 (0.5)
History of difficult intubation, No. (%)	29 (5)	29 (5)	64 (5)
Previous videolaryngoscopy, No. (%)	36 (6)	52 (8)	88 (7)
Previous awake tracheal intubation, No. (%)	5 (0.8)	15 (2)	20 (2)
Existing anaesthesia alert card, No. (%)	5 (0.8)	9 (1)	14 (1)
Mouth opening; mean (SD), cm[a]	4.3 (0.9)	4.1 (1.0)	4.2 (0.9)
Mallampati class 4, No. (%)	59 (10)	67 (10)	126 (10)
Cannot bite upper lip, No. (%)	87 (15)	92 (14)	179 (14)
Retrognathia, No. (%)	134 (22)	115 (17)	249 (19)
Neck movement < 70°, No. (%)	26 (4)	17 (3)	43 (3)
Expanding pharyngolaryngeal lesion, No. (%)	40 (7)	43 (6)	83 (7)
History of neck radiotherapy, No. (%)[b]	36 (6)	41 (6)	77 (6)
Dysphagia, No. (%)	84 (14)	83 (12)	167 (13)
Preoperative transnasal videoendoscopy, No. (%)	277 (46)	284 (42)	561 (44)
SARI score; mean (SD), 0-12 pts.	2.3 (2.0)	2.3 (1.9)	2.3 (1.9)
Wilson score; mean (SD), 0-10 pts.	1.9 (1.5)	1.8 (1.5)	1.8 (1.5)
Surgical procedures, No. (%)			
Microlaryngoscopy	75 (12)	77 (11)	152 (12)
Oropharyngeal tumor surgery[b]	24 (4)	22 (3)	46 (4)
Laryngectomy, partial or total[b]	8 (1)	6 (1)	14 (1)
Primary tracheostomy	5 (1)	6 (1)	11 (1)
Nasal surgery	91 (15)	108 (16)	199 (16)
Sinus surgery	31 (5)	33 (5)	64 (5)
Ear surgery	50 (8)	61 (9)	111 (9)
Tonsillectomy/tonsillotomy/adenoidectomy	14 (2)	18 (3)	32 (2)
Endo-/exocrine gland surgery[c]	38 (6)	27 (4)	65 (5)
Maxillofacial and neck surgery[b]	143 (24)	166 (24)	309 (24)
Lower jaw	71 (12)	95 (14)	166 (13)
Dentoalveolar	35 (6)	39 (6)	74 (6)
Others	17 (3)	22 (3)	39 (3)

ASA, ASA physical status; SARI, simplified airway risk index. [a] 3 missing values in the development and b one in the validation cohort; [b] inclusive microvascular free-flap reconstructions; nasal surgery includes: septoplasty, rhinoplasty, rhinosseptoplasty, turbinate reduction; sinus surgery includes: functional endoscopic sinus surgery, balloon sinuplasty, maxillary sinus surgery, sphenoidotomy and ethmoidectomy; ear surgery includes: tympanoplasty, stapedectomy, cochlear implantation, mastoidectomy, ossiculoplasty; endo/-exocrine gland surgery includes thyroid surgery, parathyroid surgery, transoral submandibular sialolitectomy and transoral submandibulotomy

© 2025 Popal C et al. *JAMA Otolaryngology–Head & Neck Surgery*.

eTable2 Optimal decision thresholds of the Expect-It score

Sens: sensitivity; Spez: specificity; PPV: positive predictive value; NPV: negative predictive value; the highest Youden index was found for ≥ 3 points. Further PPV and NPV were used from a utility-based perspective to determine optimal decision thresholds for the appropriate tracheal intubation technique (camera-assisted versus direct laryngoscopy; yellow marks) and appropriate tracheal intubation strategy (awake versus asleep; red marks) tracheal intubation in the development cohort. As the Expect-It tool is intended to be a diagnostic tool we considered that it should demonstrate at least a PPV of 0.5.

	**Camera-assisted intubation most appropriate**					**Awake tracheal intubation most appropriate**				
**Score**	**Sens.**	**Spec.**	**PPV**	**NPV**	**Youden**	**Sens.**	**Spec.**	**PPV**	**NPV**	**Youden**
1 pt.	0.89 [0.82-0.93]	0.58 [0.54-0.63]	0.35 [0.30-0.41]	0.95 [0.92-0.97]	0.47	1.00 [0.86-1.00]	0.51 [0.47-0.55]	0.08 [0.05-0.11]	1.00 [0.99-1.00]	0.51
2 pts.	0.72 [0.63-0.79]	0.85 [0.82-0.88]	0.56 [0.48-0.63]	0.92 [0.89-0.94]	0.57	1.00 [0.86-1.00]	0.77 [0.73-0.80]	0.15 [0.10-0.20]	1.00 [0.99-1.00]	0.77
3 pts.	0.65 [0.56-0.73]	0.94 [0.91-0.96]	0.73 [0.64-0.80]	0.91 [0.88-0.93]	0.59	1.00 [0.86-1.00]	0.85 [0.82-0.88]	0.22 [0.15-0.30]	1.00 [0.99-1.00]	0.85
4 pts.	0.54 [0.45-0.62]	0.97 [0.95-0.98]	0.81 [0.72-0.88]	0.89 [0.86-0.91]	0.51	0.92 [0.74-0.98]	0.90 [0.87-0.92]	0.27 [0.19-0.38]	1.00 [0.99-1.00]	0.81
5 pts.	0.43 [0.35-0.52]	0.98 [0.96-0.99]	0.85 [0.75-0.92]	0.87 [0.84-0.90]	0.41	0.88 [0.69-0.96]	0.93 [0.91-0.95]	0.34 [0.23-0.46]	0.99 [0.98-1.00]	0.80
6 pts.	0.34 [0.26-0.43]	0.99 [0.98-1.00]	0.93 [0.82-0.98]	0.85 [0.82-0.88]	0.34	0.75 [0.55-0.88]	0.95 [0.93-0.97]	0.40 [0.27-0.55]	0.99 [0.98-1.00]	0.70
7 pts.	0.25 [0.18-0.34]	1.00 [0.99-1.00]	1.00 [0.89-1.00]	0.84 [0.81-0.87]	0.25	0.71 [0,51- 0,85]	0.98 [0,96-0,99]	0.55 [0.38-0.71]	0.99 [0.97-0.99]	0.68
8 pts.	0.20 [0.13-0.27]	1.00 [0.99-1.00]	1.00 [0.86-1.00]	0.83 [0.80-0.86]	0.20	0.63 [0.43-0.79]	0.98 [0.97-0.99]	0.63 [0.43-0.79]	0.98 [0.97-0.99]	0.61
9 pts.	0.14 [0.09-0.21]	1.00 [0.99-1.00]	1.00 [0.82-1.00]	0.82 [0.79-0.85]	0.14	0.50 [0.31-0.69]	0.99 [0.98-1.00]	0.71 [0.47-0.87]	0.98 [0.96-0.99]	0.49
10 pts.	0.10 [0.06-0.16]	1.00 [0.99-1.00]	1.00 [0.76-1.00]	0.81 [0.79-0.84]	0.10	0.46 [0.28-0.65]	1.00 [0.99-1.00]	0.92 [0.69-0.99]	0.98 [0.96-0.99]	0.46

© 2025 Popal C et al. *JAMA Otolaryngology–Head & Neck Surgery*.

eFigure 2. Histogram showing the distribution of the Expect-It score (range 0 – 23 points) in the validation cohort (n=680).

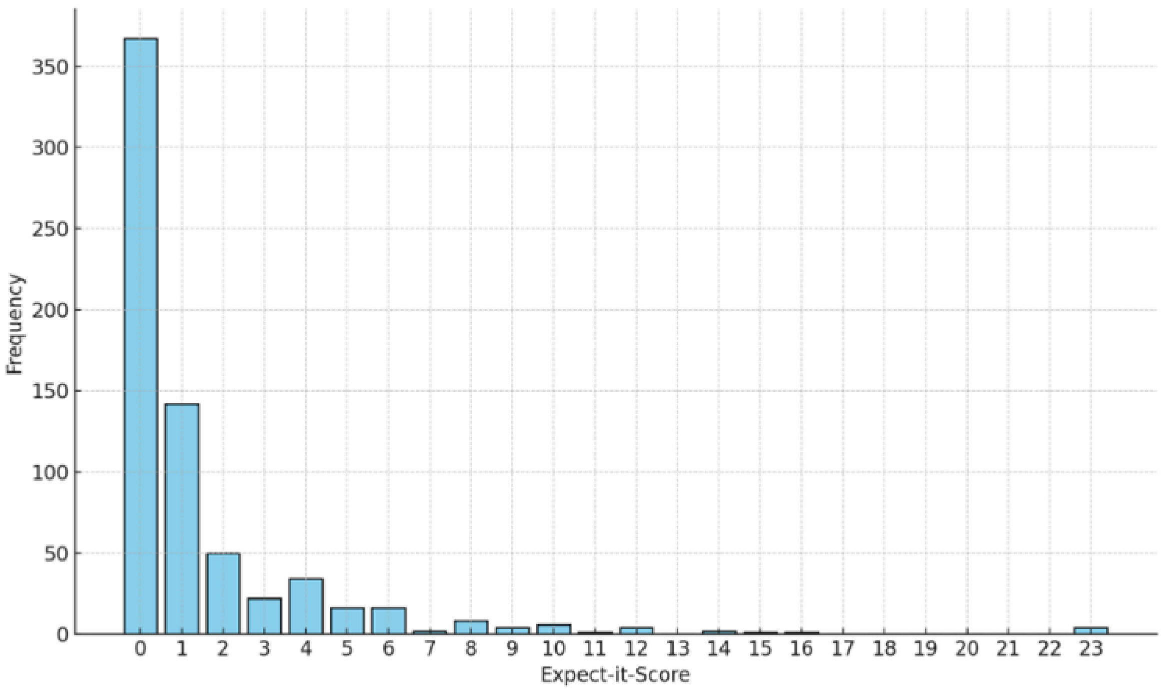

eFigure 3. Calibration belts illustrating the agreement between the observed and predicted probabilities for the appropriate tracheal intubation technique (left) and strategy (right) in the development cohort. The red diagonal line represents perfect agreement, with 80% (light grey) and 95% (dark grey) confidence intervals.

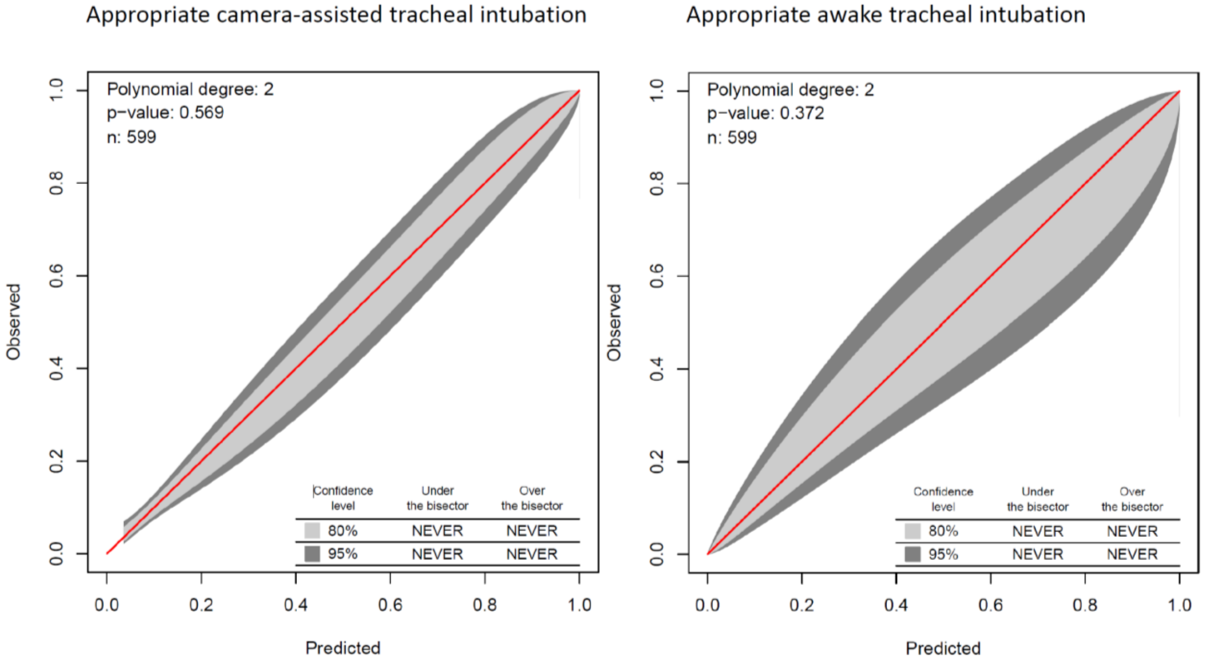

© 2025 Popal C et al. *JAMA Otolaryngology–Head & Neck Surgery*.

eTable 3. Accuracy of preoperative decision-making (preoperatively proposed tracheal intubation technique and strategy) to predict the appropriate tracheal intubation technique and strategy of Experienced-based (nonalgorithm-based) decisions (development cohort) and Expected-it based decisions (validation cohort)

	**Appropriate technique and strategy**	
**Proposed first-line technique and strategy**	**Sensitivity (%)**	**Specificity (%)**
**Development cohort (n=602) – nonalgorithm-based decision-making**		
Camera-assisted tracheal intubation	35 (27-44)	96 (93-97)
Awake tracheal intubation	29 (15-50)	98 (97-99)
**Validation cohort (n=680) – Expect-It based decision-making**		
Camera-assisted tracheal intubation	88 (81-93)	97 (96-98)
Awake tracheal intubation	97 (81-100)	100 (99-100)

© 2025 Popal C et al. *JAMA Otolaryngology–Head & Neck Surgery*.

eReferences

1. Apfelbaum JL, Hagberg CA, Connis RT, et al. 2022 American Society of Anesthesiologists Practice Guidelines for Management of the Difficult Airway. *Anesthesiology.* 2022;136(1):31-81.
2. Law JA, Duggan LV, Asselin M, et al. Canadian Airway Focus Group updated consensus-based recommendations for management of the difficult airway: part 2. Planning and implementing safe management of the patient with an anticipated difficult airway. *Can J Anaesth.* 2021;68(9):1405-1436.
3. Rosenblatt WH, Yanez ND. A Decision Tree Approach to Airway Management Pathways in the 2022 Difficult Airway Algorithm of the American Society of Anesthesiologists. *Anesth Analg.* 2022;134(5):910-915.
4. Barclay-Steuart A, Grosshennig HL, Sasu P, et al. Transnasal Videoendoscopy for Preoperative Airway Risk Stratification: Development and Validation of a Multivariable Risk Prediction Model. *Anesth Analg.* 2023;136(6):1164-1173.
5. Sasu PB, Pansa JI, Stadlhofer R, et al. Nasendoscopy to Predict Difficult Videolaryngoscopy: A Multivariable Model Development Study. *J Clin Med.* 2023;12(10).
6. Köhl V, Wünsch VA, Müller MC, et al. Hyperangulated vs. Macintosh videolaryngoscopy in adults with anticipated difficult airway management: a randomised controlled trial. *Anaesthesia.* 2024;79:957-966.
7. Kohse EK, Siebert HK, Sasu PB, et al. A model to predict difficult airway alerts after videolaryngoscopy in adults with anticipated difficult airways - the VIDIAC score. *Anaesthesia.* 2022;77(10):1089-1096.
8. Wünsch VA, Köhl V, Breitfeld P, et al. Hyperangulated blades or direct epiglottis lifting to optimize glottis exposure in difficult Macintosh videolaryngoscopy: A non-inferiority analysis of a prospective observational study. *Front Med (Lausanne).* 2023;10:1292056.
9. Cook TM. A new practical classification of laryngeal view. *Anaesthesia.* 2000;55(3):274-279.
10. Yentis SM, Lee DJ. Evaluation of an improved scoring system for the grading of direct laryngoscopy. *Anaesthesia.* 1998;53(11):1041-1044.
11. Cormack RS. Laryngoscopy grades. *Anaesthesia.* 1999;54(9):911-912.
12. Siebert HK, Kohse EK, Petzoldt M. A universal classification for videolaryngoscopy using the VIDIAC score requires real world conditions: a reply. *Anaesthesia.* 2023;78(1):126.
13. Cormack RS, Lehane J. Difficult tracheal intubation in obstetrics. *Anaesthesia.* 1984;39(11):1105-1111.
14. Sasu PB, Gutsche N, Kramer R, et al. Universal paediatric videolaryngoscopy and glottic view grading: a prospective observational study. *Anaesthesia.* 2024;79(10):1062-1071.

© 2025 Popal C et al. *JAMA Otolaryngology–Head & Neck Surgery*.

15. Dohrmann T, Gutsche N, Kramer R, et al. Prospective development and validation of a universal classification for paediatric videolaryngoscopic tracheal intubation: the PeDiAC score. *Anaesthesia.* 2024;79(11):1201-1211.
16. Samsoon GL, Young JR. Difficult tracheal intubation: a retrospective study. *Anaesthesia.* 1987;42(5):487-490.
17. Detsky ME, Jivraj N, Adhikari NK, et al. Will This Patient Be Difficult to Intubate?: The Rational Clinical Examination Systematic Review. *JAMA.* 2019;321(5):493-503.
18. Wilson ME, Spiegelhalter D, Robertson JA, Lesser P. Predicting difficult intubation. *Br J Anaesth.* 1988;61(2):211-216.
19. Hastie T, Tibshirani R, Tibshirani R. Best Subset, Forward Stepwise or Lasso? Analysis and Recommendations Based on Extensive Comparisons. *Statist Sci.* 2020;35(4):579-592.
20. Ullmann T, Heinze G, Hafermann L, Schilhart-Wallisch C, Dunkler D, for TGotSi. Evaluating variable selection methods for multivariable regression models: A simulation study protocol. *PLoS One.* 2024;19(8):e0308543.
21. Grensemann J, Mohlenkamp E, Breitfeld P, et al. Tracheal Tube-Mounted Camera Assisted Intubation vs. Videolaryngoscopy in Expected Difficult Airway: A Prospective, Randomized Trial (VivaOP Trial). *Front Med (Lausanne).* 2021;8:767182.
22. Petzoldt M, Engels Y, Popal Z, et al. Elective Tracheal Intubation With the VieScope-A Prospective Randomized Non-inferiority Pilot Study (VieScOP-Trial). *Front Med (Lausanne).* 2022;9:820847.
23. Avidan A, Shapira Y, Cohen A, Weissman C, Levin PD. Difficult airway management practice changes after introduction of the GlideScope videolaryngoscope: A retrospective cohort study. *Eur J Anaesthesiol.* 2020;37(6):443-450.
24. Dawson SR, Taylor L, Farling P. The true cost of videolaryngoscopy may be trainee experience in fibreoptic intubation. *Br J Anaesth.* 2015;115(1):134-135.
25. Thomas G, Kelly F, Cook T. Introduction of videolaryngoscopy has not reduced rates of fibreoptic intubation. *Br J Anaesth.* 2016;116(5):717.
26. Thomas G, Kelly F, Cook T. No reduction in fibreoptic intubation rates with universal video laryngoscopy. *Can J Anaesth.* 2016;63(1):113.
27. Arne J, Descoins P, Fusciardi J, et al. Preoperative assessment for difficult intubation in general and ENT surgery: predictive value of a clinical multivariate risk index. *Br J Anaesth.* 1998;80(2):140-146.
28. Ayuso MA, Sala X, Luis M, Carbo JM. Predicting difficult orotracheal intubation in pharyngo-laryngeal disease: preliminary results of a composite index. *Can J Anaesth.* 2003;50(1):81-85.
29. Glimm E, Maurer W, Bretz F. Hierarchical testing of multiple endpoints in group-sequential trials. *Stat Med.* 2010;29(2):219-228.

© 2025 Popal C et al. *JAMA Otolaryngology–Head & Neck Surgery*.

30. Hung HM, Wang SJ, O'Neill R. Statistical considerations for testing multiple endpoints in group sequential or adaptive clinical trials. *J Biopharm Stat.* 2007;17(6):1201-1210.
31. Li Y, Ghosh D. A two-step hierarchical hypothesis set testing framework, with applications to gene expression data on ordered categories. *BMC Bioinformatics.* 2014;15:108.
32. Luo L, Kang H, Li X, Ness SA, Stidley CA. Two-step mixed model approach to analyzing differential alternative RNA splicing. *PLoS One.* 2020;15(10):e0232646.
33. Donkers H, Graff M, Vernooij-Dassen M, Nijhuis-van der Sanden M, Teerenstra S. Reducing sample size by combining superiority and non-inferiority for two primary endpoints in the Social Fitness study. *J Clin Epidemiol.* 2017;81:86-95.
34. Berger RL. Multiparameter hypothesis testing and acceptance sampling. *Technometrics.* 1982;24:295-300.
35. Tamhane AC, Logan BR. A superiority-equivalence approach to one-sided tests on multiple endpoints in clinical trials. *Biometrika.* 2004;91 (3):715-727.
36. Butcher NJ, Monsour A, Mew EJ, et al. Guidelines for Reporting Outcomes in Trial Reports: The CONSORT-Outcomes 2022 Extension. *JAMA.* 2022;328(22):2252-2264.

© 2025 Popal C et al. *JAMA Otolaryngology–Head & Neck Surgery*.
