# Supplementary material for: Decision-Making Tool for Planning Camera-Assisted and Awake Intubation in Head and Neck Surgery
Source: JAMA Otolaryngol Head Neck Surg. 2025 May 1;151(6):585–94. doi: 10.1001/jamaoto.2025.0538 (PMC12046521; doi:10.1001/jamaoto.2025.0538)
Supplement: Supplement 2. — Data Sharing Statement [file jamaotolaryngolheadnecksurg-e250538-s002.pdf]

Data Sharing Statement

Popal. Decision-Making Tool for Planning Camera-Assisted and Awake Intubation in Head and Neck Surgery. *JAMA Otolaryngol Head Neck Surg*. Published May 01, 2025. doi:10.1001/jamaoto.2025.0538

Data
Data available: Yes
Data types: Deidentified participant data
How to access data: Deidentified participant data can be accessed through the contact with the corresponding author (m.petzoldt@uke.de).
When available: With publication

Supporting Documents
Document types: None

Additional Information
Who can access the data: Researchers whose proposed use of the data has been approved
Types of analyses: Research that relies on systematic data synthesis, e.g. systematic literature review, meta-analysis etc. with permission
Mechanisms of data availability: With investigator support
